# The exercise-induced inflammatory response in inflammatory bowel disease: A systematic review and meta-analysis

**DOI:** 10.1371/journal.pone.0262534

**Published:** 2022-02-04

**Authors:** Kelly A. Baker, Timothy D. Miller, Frank E. Marino, Tegan E. Hartmann

**Affiliations:** School of Allied Health, Exercise and Sport Sciences, Charles Sturt University, Bathurst, New South Wales, Australia; Poznan University of Physical Education, POLAND

## Abstract

**Background:**

This study investigated selected inflammatory responses to acute and chronic exercise in individuals with inflammatory bowel disease (IBD).

**Methods:**

A systematic review and meta-analysis was conducted on all relevant exercise-based intervention publications with IBD participants. The study included articles that utilised a broad range of acute and chronic exercise interventions, with inflammatory biomarkers measured and symptoms documented, both pre- and post-exercise for those with IBD. The search was limited to studies published in English, the use of human participants, and primary studies, with no restrictions on date of publication or participant’s age. Articles were retrieved through the electronic databases: PubMed, SPORTDiscus, and Scopus. This study adhered to Preferred Reporting Items for Systematic Reviews and Meta-Analysis (PRISMA) guidelines.

**Results:**

Six inflammatory markers were included in the meta-analysis which consisted of five studies. Exercise interventions resulted in no significant difference in IL-6 (*SMD* = -0.09; 95% *CI* = -0.49, 0.30; *P* = 0.64), TNF-α (*SMD* = 0.08; 95% *CI* = -0.31, 0.48; *P* = 0.68), CRP (*SMD* = -0.04; 95% *CI* = -0.58, 0.50; *P* = 0.89), IL-17 (*SMD* = 0.15; 95% *CI* = -0.45, 0.76; *P* = 0.62), leukocytes (*SMD* = 0.40; 95% *CI* = -0.53, 1.33; *P* = 0.40) or lymphocytes (*SMD* = 0.32; 95% *CI* = -0.33, 0.97; *P* = 0.33), thus, indicating exercise may have no effect on inflammatory markers in IBD. Bowel symptoms improved following regular moderate exercise that incorporated stress management.

**Conclusion:**

Heterogeneity among the identified literature may have led to exercise interventions being ineffective in reducing inflammation. Although the limited number of eligible studies may reduce the reliability of results, it emphasises the need for additional research in this domain. Importantly, no adverse symptomatic responses to exercise indicate that exercise is safe for IBD patients.

## Introduction

Inflammatory bowel disease (IBD) is an idiopathic, chronic, gastrointestinal (GI) tract disorder characterised pathologically by intestinal inflammation [1,2]. Crohn’s disease (CD) and ulcerative colitis (UC) are primary inflammatory intestinal conditions and are the most rigorously studied types of IBD due to their higher prevalence [3,4]. The aetiology of IBD is relatively unknown; however, a complex interaction of genetics, environmental factors, mucosal immune system dysfunction, and alterations in intestinal microbiota are suggested to contribute to the development and progression of IBD [5–8]. Additional factors, such as the impact of the dysregulated gut-brain axis, have been acknowledged to contribute to the chronic inflammation observed in the disease [9,10], with specific physiological mechanisms identified to increase the intestinal inflammation. This includes loss of equilibrium between the hypothalamic-pituitary-adrenocortical (HPA) axis and the limbic system [11] as well as an imbalance of the sympathetic-parasympathetic nervous systems [12]. The primary source of the disruption is unknown as the integrated interaction is not completely understood.

The unknown pathogenic mechanisms of IBD and the physiological characteristics of intestinal inflammation has led CD and UC to be identified as an immune-mediated inflammatory disease [13–15]. Inflammatory cytokines in the GI tract have a direct involvement in the pathogenesis and exacerbation of IBD, either through an excessive production of pro-inflammatory cytokines or ineffective production of anti-inflammatory cytokines [1,16]. Specifically, IBD results in immunological and physiological changes through the irregular activation of inflammatory cytokines. This consequently results in an imbalance of cytokines in the local mucosa which leads to intestinal inflammation, symptoms, and increased frequency of active disease states [1,17]. In IBD, specific imbalances of cytokines include an influx of pro-inflammatory cytokine tumour necrosis factor-alpha (TNF-α) [5,16], as well as a decrease of the anti-inflammatory cytokine IL-1 receptor antagonist (IL-1ra) [18] and interleukin (IL) -10 [19,20], whilst an increase of pro-inflammatory cytokine IL-5 is observed in UC [5,16]. Additionally, the speculated pathological significance of pro-inflammatory IL-17 transpires as it upregulates pro-inflammatory cytokines TNF-α, IL-1 beta (IL-1β), and IL-6. When IL-6 proceeds the release of TNF-α it possesses pro-inflammatory properties and promotes local and systemic inflammation [21] which contributes to the chronic inflammatory process in IBD [22]. Similarly, CRP is another known contributor to overall inflammation and is a well-established marker for IBD [23,24]; whilst leukocytes and lymphocytes are associated with the debilitating inflammatory process leading to inflammatory diseases and potentially intestinal inflammation [25,26]. Further immunological and neuroendocrine changes, influenced through a series of inflammatory responses elicited by stress, contributes to the intestinal inflammation, and consequently disease exacerbation [27,28].

The dysregulation of inflammatory cytokines in the GI tract contributes to the progressive and destructive manifestation of IBD [1,16]. It is suggested that the level of intestinal inflammation governs the course of the disease and is the primary determinant of whether the disease in an active or quiescent state at any given time [5]. The unpredictable active states are characterised by severe symptomatic periods and extra-intestinal manifestations including abdominal pain, diarrhoea, and fatigue [5,29]; whilst quiescent states or clinical remission represents a period of reduced inflammation, disease activity and associated symptoms [5,16]. Recently, it has been suggested that the severity of IBD symptoms has intensified and diagnosis occurs earlier in life [30]. These factors have contributed to adverse effects on patient’s health-related quality of life (HRQoL) [31,32]. Currently, there is no known cure for IBD and treatment modalities, such as diet modification [33] and faecal microbiota transportation [34], remain incompletely effective [5,31]. Accordingly, alternative mechanisms to manage the condition warrant investigation [30,35].

Evidence suggests that acute exercise induces a cascade of anti-inflammatory cytokines, specifically initiated through the exponential increase of muscle-derived IL-6, and with regular training, may lower basal levels of circulating inflammatory markers and reduce chronic low-grade inflammation [36–38]. Despite the pathological significance pro-inflammatory IL-6 has on the chronic inflammatory process in IBD [1,5,22], the production of IL-6 in skeletal muscle as a response to exercise is likely to be anti-inflammatory and associated with the beneficial role of exercise-related metabolic changes [39]. The synthesis of muscle-derived IL-6 precedes the cascade of potent anti-inflammatory cytokines IL-10 and cytokine inhibitor IL-1ra to produce an anti-inflammatory environment [40]. Specifically, IL-10 and IL-1ra reduces signal transduction to inhibit pro-inflammatory TNF-α and IL-1β [41], whilst IL-6 further exerts inhibitory effects on lipopolysaccharide induced TNF-α production [37], as well as stimulating the release of soluble tumour necrosis factor receptors which acts as a natural inhibitor for TNF-α [42,43]. The prevention or attenuation of inflammation has been identified to be a likely important mechanism to protect against the development of chronic diseases [44]. Such reductions in systemic inflammation have been observed in apparently healthy individuals as well as chronic conditions such as cardiovascular disease (CVD), type 2 diabetes mellitus (T2DM), breast cancer, and colon cancer [37,43], however, it is unknown whether such reductions occur in IBD. Despite the ambiguity around the exercise-induced inflammatory response in IBD, evidence has emerged to support a positive relationship between moderate-to-vigorous intensity physical activity and improved HRQoL for IBD patients [32], assuming a reduction in systemic inflammation is associated with an increased HRQoL rating. However, limited research has confirmed the independent distinction between systemic inflammation and HRQoL, therefore further investigation is warranted.

Exercise has the potential to attenuate cytokine imbalances through the inflammatory pathways induced by acute bouts of exercise and regular training [37]. The use of exercise as an alternative or complementary treatment strategy for patients with IBD has been acknowledged [31,45,46]. However, there is currently insufficient evidence to either support or refute the use of exercise as an adjunct treatment modality for IBD. Accordingly, elucidating the acute and chronic exercise-induced pro- and anti-inflammatory responses in IBD may provide a greater rationale for implementation of exercise as a management strategy.

The purpose of this meta-analysis was to investigate the anti-inflammatory response induced from acute and chronic exercise in IBD. We also aimed to explore the impacts exercise might have on disease activity and associated symptoms and examined the effect of different exercise modalities on the inflammatory response for people with IBD. We hypothesised that in response to acute and chronic exercise the pro-inflammatory markers in IBD would decrease, resulting in attenuated disease activity and associated symptoms. Additionally, moderate-intensity aerobic exercise is hypothesised to result in the greatest decrease in pro-inflammatory markers, augmenting the anti-inflammatory response through exercise.

## Materials and methods

### Protocol and registration

The protocol outlined below was established *a priori* in compliance with the identified specifications in the Preferred Reporting Items of Systematic Reviews and Meta-Analysis Protocols (PRISMA) [47,48] (S1 Table). Further, this review was registered at the International Prospective Register for Systematic Reviews (PROSPERO): registration number CRD42020191279.

### Data acquisition and study selection

Studies that examined the exercise-induced inflammatory response in individuals with IBD were analysed in the meta-analysis. Studies identified for potential inclusion were retrieved through a systematic search of PubMed, SPORTDiscus, and Scopus, with use of the search terms and Boolean operators “inflammatory bowel disease OR Crohn’s disease OR ulcerative colitis” AND “inflammation OR inflammatory markers OR biomarkers OR cytokines OR inflammatory response” AND “exercise OR physical activity OR exercise intervention OR exercise protocol OR cycling”. The search was limited to studies published in English, the use of human participants, and primary studies. The initial literature search commenced May 26^th^ 2020 and the final literature search was completed on August 31^st^ 2020.

**Fig 1:**
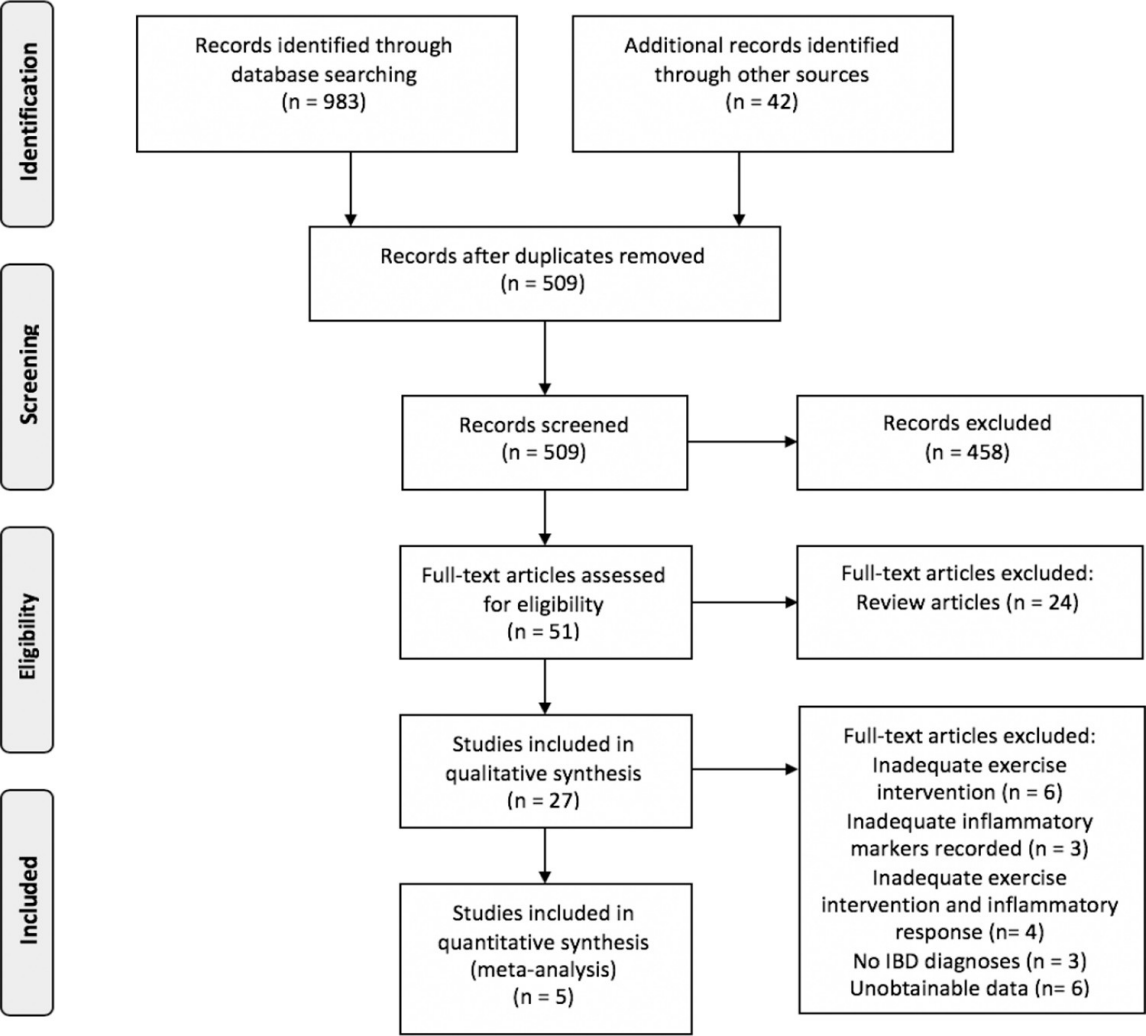
PRISMA (Preferred Reporting Items of Systematic Reviews and Meta–Analysis Protocols) flow diagram. Overview of the identification, screening, eligibility, and inclusion process for the quantitative review.

The initial database search of literature identified a total of 983 potential studies; an additional 42 articles were identified through an extensive manual search of Google Scholar. All recognised citations were uploaded to a reference manager software (EndNote X9.3.3), where duplicates were removed. Following the removal of duplicates, titles and abstracts were screened for suitability. The eligibility phase began with a search of each reference list from the identified articles for further potential pertinent citations for the present review. A full detailed outline of the search protocol applied in the review is depicted in Fig 1. The studies were further processed through an eligibility criterion to be considered for the meta-analytical procedure. For inclusion, potential studies were required to meet the following inclusion criteria: (1) patients must be diagnosed with a form of IBD (CD or UC); (2) an exercise intervention must have been applied as a treatment; (3) pro- and anti-inflammatory markers must have been reported pre- and post-exercise intervention; and (4) disease activity must have been reported pre- and post-exercise intervention. Studies were excluded if they did not adhere to the inclusion criteria, were secondary or review studies, included patients diagnosed with other comorbidities, or if they were animal studies. The search terms and eligibility criteria utilised was determined by all authors (KB, TH, TM, FM), before study selection was completed in duplicate (KB, TH).

### Data synthesis and analysis

Prior to extraction of data, the studies which met the inclusion criteria were assessed for suitability. For inclusion, the identified exercise intervention had to be classified or assumed as low-, moderate-, or high-intensity. Table 1 lists the exercise interventions used and inflammatory markers investigated; the pertinent inflammatory markers analysed were derived from the eligible studies through consensus among authors with no discrepancies. The data of the inflammatory markers recorded, pre- and post- exercise intervention, were extracted; the means and corresponding standard deviations (*SD*) were removed directly. Data that were expressed in median and interquartile range or mean and standard error of the mean (*SEM*), the number of participants, exact *p*-value and recognised statistical comparative test were extracted. If data could not be obtained from the included studies with use of the methods presented, extrapolation through digitisation of figures were completed to calculate the mean and *SD* (GraphClick v2.9 beta 2). If data were still unable to be extracted following extrapolation, the corresponding author was contacted. If contacted authors were unable to provide required data necessary for the meta-analysis, the study was excluded from the analysis. All outcome inflammatory markers required for data analysis were catalogued into a Microsoft Excel spreadsheet (Microsoft Office v16.16.25) by one author (KB), secondary assessments were then completed by three co-authors (TH, TM, FM) before data were analysed.

**Table 1:** Exercise intervention included and inflammatory markers investigated with their corresponding authors.

Author (Year)	Exercise intervention	Inflammation markers recorded
Cronin et al. (2019) [45]	Combined aerobic and resistance training; couch to 5km training program and study-specific resistance training machines.	TNF-α IL-6 IL-8 IL-10 CRP
Elsenbruch et al. (2005) [60]	Structured and supervised training program (stress management, moderate exercise, moderate Mediterranean diet, cognitive behavioural techniques).	Leukocytes Lymphocytes Granulocytes Monocytes GH
Klare et al. (2015) [31]	Supervised outdoor running; designed from a running program for untrained people.	Leukocytes CRP Calprotectin
Ploeger et al. (2012) [46]	Structured, laboratory setting; one preliminary session and two cycling exercise interventions with one week between (MICE and HIIE). MICE: cycled at 50% of their determined Wpeak. HIIE: cycled at 100% of their determined Wpeak.	Leukocytes Lymphocytes Neutrophils Monocytes GH IGH TNF-α IL-6 IL-17
Sharma et al. (2015) [61]	Supervised yoga intervention followed by at home daily practice; comprised of physical postures, pranayama and meditation.	ECP sIL-2R

sIL–2R, soluble interleukin–2 receptor; IL–6, interleukin–6; IL–8, interleukin–8; IL–10, interleukin–10; IL–17, interleukin–17; CRP, C–reactive protein; TNF–α, tumour necrosis factor–alpha; ECP, eosinophilic cationic protein; GH, growth hormone; IGH, insulin–like growth hormone; MICE, moderate–intensity continuous exercise; HIIE, high–intensity interval exercise; Wpeak, peak aerobic mechanical power.

Confirmed outcome data expressed as mean and *SD* were imported into a statistical software (R-studio v1.3.959) and calculated directly for Hedges’ *g* effect size. The remaining data were transferred into an online effect size calculator [49] where Cohen’s *d* was calculated in respect to the recognised statistical comparative test, prior to the import and calculation of Hedges’ *g* in statistical software (R-studio v1.3.959). Hedges’ *g* calculation was the preferred effect size to minimise a potential bias from small sample sizes which Cohen’s *d* can produce [50]. Furthermore, Hedges’ *g* was expressed as standardised mean difference (*SMD*) with small, moderate, and large standardised conventions, whereby values of 0.2, 0.5, and 0.8 respectively, determined the magnitude of the effect [51]. In addition, the DerSimonian and Laird [52] (DL) random-effect model was completed with use of the pre-calculated effect size data in the statistical software (R-studio v1.3.959) which required Hedges’ *g* effect size rather than Cohen’s *d*. Furthermore, the publication bias was investigated through the graphical representation of a funnel plot, with the funnel plot asymmetry further statistically tested with the use of Egger’s test of intercept [53]. If significant publication bias was detected, Duval and Tweedie’s trim and fill procedure was applied [54], and the resultant effects on Hedges’ *g* and the 95% confidence interval (*CI*) was explored.

### Methodological quality and risk of bias assessment

The 11 item Physiotherapy Evidence Database (PEDro) scale was used for the assessment of an individual study’s methodological quality and risk of bias [55]; this includes random allocation, concealed allocation, comparability of groups at baseline, blinding of subjects, therapists, and assessors, analysis by intention to treat, completeness of follow-up as well as between-group statistical comparison and reports. The internal validity and interpretability criteria are useful for assessing methodological quality [56]. Evidence has shown a bias, provided by low-quality studies, effects the treatment effectiveness [48,57,58]; Teasell et al. [59] classified the articles as follows: *excellent*: 9–10, *good*: 6–8, *fair*: 4–5, and *poor*: < 4. Exercise interventions do not allow for blinding of subjects and therapists. It is recognised that in the evaluation of complex interventions, such as exercise programs, the highest possible score is 8/10. Consistent with the PEDro scale scoring method, each criterion was rated “yes” or “no” where applicable. The summary score was then determined simply by counting the number of items which were satisfied in the article (with the exclusion of item 1, eligibility criteria and source). Therefore, the total can range from 0 (*low quality*) to 10 (*high quality*) out of 10 [55]. Articles are displayed in descending order, with high quality studies at the top of the list. Each author independently reviewed all citations with any discrepancies settled by consensus.

## Results

### Study characteristics

A total of 5 peer-reviewed studies met the inclusion criterion and were assessed for the review [31,45,46,60,61]. Ploeger et al. [46] reported data on two independent exercise interventions, thus, both data sets were extracted and analysed independently. Additionally, to identify the different exercise protocols, moderate-intensity continuous exercise (MICE) was referred to as *Ploeger (MICE)* and high-intensity intermittent exercise (HIIE) was referred to as *Ploeger (HIIE)*. The five studies combined resulted in 192 male and female participants with a diagnosis of IBD. Four studies completed the research on IBD patients greater than 16 years of age [31,45,60,61], with Elsenbruch et al. [60] exclusively recruiting UC patients; whilst one study focused on paediatric CD with the average age of 14.5 ± 2.4 years [46]. There was marked variance in the duration of, and modalities used within, the identified exercise interventions. Ploeger et al. [46] investigated an acute cycling bout, whereas the remainder of the studies completed chronic exercise interventions (9 weeks ± 1 week) with either 3-times per week [31,45] or 60–80 hours of total exercise duration [60,61].

The effects of exercise on disease activity and symptoms were measured through a variety of IBD specific questionnaires pre- and post- exercise intervention. Elsenbruch et al. [60] reported significant improvements in Inflammatory Bowel Disease Questionnaire (IBDQ) bowel symptoms component following the regular exercise intervention utilised, which contained components of stress management and moderate-intensity exercise, while four of the five studies reported no significant symptomatic responses [31,45,46,61]. Further, at a non-significant level, Sharma et al. [61] noted the control group experienced more intestinal colicky pain whilst the intervention group reported less arthralgia. Similarly, a non-significant decrease in Crohn’s Disease Activity Index (CDAI) and Racmilewitz Index (RI) was observed following a 10-week moderate-intensity aerobic exercise intervention [31]. Exercise was reported as safe following an 8-week aerobic and resistance training intervention [45] as well as after both acute MICE and HIIE exercise protocols [46] as disease exacerbation did not occur. It is noted that three of the five studies used *clinical remission* as an inclusion criterion [45,46,61], one included *clinical remission* or *mildly active disease* [31], and the other reported that 73% of patients were in *clinical remission* [60]. A detailed overview of characteristics of included studies are reported in Table 2.

**Table 2:** Characteristics of Included Studies.

Studies	Subjects and study design	Medication	Activity monitored	Physical activity levels	Exercise intervention	Duration, frequency, and intensity	Inflammation markers recorded	Primary outcome	Secondary findings
Cronin et al. (2019) [45]	17 IBD patients, aged 18–40, clinical remission; randomised, cross -over trial.	Disease maintenance therapy; 15% on anti-TNF-α therapy.	HBI and Simple Colitis Index	Physically inactive or have low levels of activity (defined by the IPAQ). No involvement in regular or organised exercise a month prior to recruitment.	Combined aerobic and resistance training; couch to 5km training program and study-specific resistance training machines.	3-times per week for 8-weeks; moderate-intensity.	IL-6 IL-8 IL-10 TNF-α CRP	Similar levels, pre- and post-exercise intervention, were observed for pro-inflammatory markers (IL-6, IL-8, IL-10, TNF-α and CRP).	No significant differences, deterioration or improvements, occurred to disease activity scores post exercise intervention.
Elsenbruch et al. (2005) [60]	30 UC patients, aged 18–65, low-disease activity or clinical remission; prospective, randomised, waiting-control study design.	Patients in use of medication (immunosuppressants and corticosteroids >10mg/day) were excluded.	CAI and IBDQ	Regular exercise prior to the exercise intervention was completed by 8/15 in the intervention group and 8/15 in the control group.	Structured and supervised training program (stress management, moderate exercise, Mediterranean diet, cognitive behavioural techniques).	60-hours over a 10-week period (6-hours, one day a week).	Leukocytes Lymphocytes Granulocytes Monocytes GH	No significant group differences, at baseline or in response to therapy, in lymphocyte subsets.	After the exercise intervention, statistically significant improvements were shown on the IBDQ scale Bowel Symptoms (*P* < 0.01) compared to the control group.
Klare et al. (2015) [31]	30 IBD patients, aged > 18, mildly active disease or remission; block-randomised study.	Participants (*n* = 26) continued their IBD specific drugs.	CDAI or RI	Participants who participated in regular structured physical activity (>2 hrs/week) were excluded. Physical activity levels were not specified.	Supervised outdoor running; designed from a running program for untrained people.	3-times per week for 10-weeks; moderate-intensity.	Leukocytes CRP Calprotectin	A statistically significant decrease in leukocytes pre- and post-exercise intervention (7.0 ± 2.2 vs. 5.6 ± 1.5, *P* = 0.016). Calprotectin levels increased (mean increase 185.0 ± 324.8mg/kg, *P* = 0.062) however, was not statistically significant from zero, pre- and post-exercise intervention. No reported change for CRP levels.	A decrease in CDAI and RI was observed from pre- and post-exercise intervention, however, no statistically significant was achieved (*P* = 0.372 & 0.266, respectively).
Ploeger et al. (2012) [46]	15 CD youth patients matched with 15 healthy participants.	Medication (5-ASA *n* = 13, azathioprine *n* = 4, methotrexate *n* = 2, infliximab *n* = 1) was continued.	PCDAI	Permitted to maintain normal exercise activity (not specified). Strenuous exercise was refrained from 24hrs prior to each experimental visit.	Structured, laboratory setting; one preliminary session and two cycling exercise interventions with a week between (MICE and HIIE). MICE: cycled at 50% of their determined Wpeak. HIIE: cycled at 100% of their determined Wpeak.	MICE: Two cycling bouts of 30-mins, 6-mins rest between bouts. HIIE: 6 sets of 15-second bouts repeated 4 times; 1-min rest between bout and 6-mins rest between sets.	Leukocytes Lymphocytes Neutrophils Monocytes GH IGF TNF-α IL-6 IL-17	No significant difference in TNF-α in CD patients, pre- and post-, MICE and HIIE; healthy control reported a significant change post- MICE (*P* < 0.05). IL-6 significantly increased for patients at exercise-end in MICE, however, returned to baseline levels by 60-mins post; healthy controls reported similar increase however elevation was maintained 60-mins post.	Concluded exercise probably does not exacerbate inflammation.
Sharma et al. (2015) [61]	100 IBD patients (UC: *n* = 60, CD: *n* = 40), aged 16–60, clinical remission phase; parallel-arm, randomised study.	Medical treatment continued throughout intervention.	Truelove and Witts (1955) and CDAI	Participants that practiced yoga within at least one year preceding the study were excluded. Physical activity levels were not specified.	Supervised yoga intervention followed by at home daily practice; comprised of physical postures, pranayama and meditation.	1-hour daily for 8-weeks; non-specific intensity, assumed low-intensity.	ECP sIL-2R	No statistically significant change occurred pre- and post-exercise intervention for UC and CD (*P* > 0.05).	Symptoms showed no statistically significant change pre- and post-exercise intervention. Fewer participants in the intervention reported arthralgia, whilst more control participants reported colicky pain.

CD, Crohn’s Disease; UC, ulcerative colitis; IBD, inflammatory bowel disease; HBI, Harvey’s Bradshaw Index; CAI, Colitis Activity Index; IBDQ, Inflammatory Bowel Disease Questionnaire; CDAI, Crohn’s Disease Activity Index; RI, Rachmilewitz Index; PCDAI, Paediatric Crohn’s Disease Activity Index; ASA, aminosalicylate; sIL–2R, soluble interleukin–2 receptor; IL–6, interleukin–6; IL–8, interleukin–8; IL–10, interleukin–10; IL–17, interleukin–17; CRP, C–reactive protein; TNF–α, tumour necrosis factor–alpha; ECP, eosinophilic cationic protein; GH, growth hormone; IGH, insulin–like growth hormone; MICE, moderate–intensity continuous exercise; HIIE, high–intensity interval exercise; Wpeak, peak aerobic mechanical power; min/s, minute/s.

### Risk of bias

Three of the five studies assessed were considered randomised controlled trials (RCT) [31,45,61]. One study was excluded as RCT due to three participants having preference [60]. Further, Ploeger et al. [46] was identified as a match-control study. All studies involved an exercise intervention; therefore, items 5 & 6 of the PEDro scale did not receive a point, thus, the highest possible result was reduced to 8/10. The risk of bias criteria was reported as poor, fair, good, or excellent, and provided decisions for justification. All PEDro scores were > 4, thus all studies remained in the analysis. The average PEDro score of all 5 trials was 5.6 ± 1.04. Table 3 displays the reported PEDro scores and corresponding studies.

**Table 3:** Risk of bias.

Study	Item 2	Item 3	Item 4	Item 5	Item 6	Item 7	Item 8	Item 9	Item 10	Item 11	Total
Sharma et al. 2015 [61]	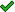	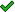	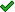	✖	✖	✖	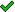	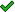	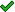	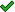	7/10
Cronin et al. 2019 [45]	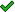	✖	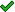	✖	✖	✖	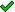	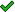	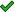	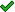	6/10
Klare et al. 2015 [31]	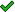	✖	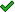	✖	✖	✖	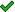	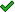	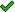	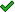	6/10
Elsenbruch et al. 2005 [60]	✖	✖	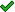	✖	✖	✖	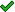	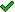	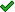	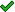	5/10
Ploeger et al. 2012 [46]	✖	✖	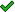	✖	✖	✖	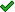	✖	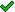	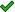	4/10

### Publication bias

Publication bias was not assessed because there were five studies included in this review, with a maximum of four studies included in the meta-analysis due to inconsistencies with inflammatory markers measured. The minimum number of studies required to perform a funnel plot for publication bias is ten [62].

### Meta-analysis

**Fig 2:**
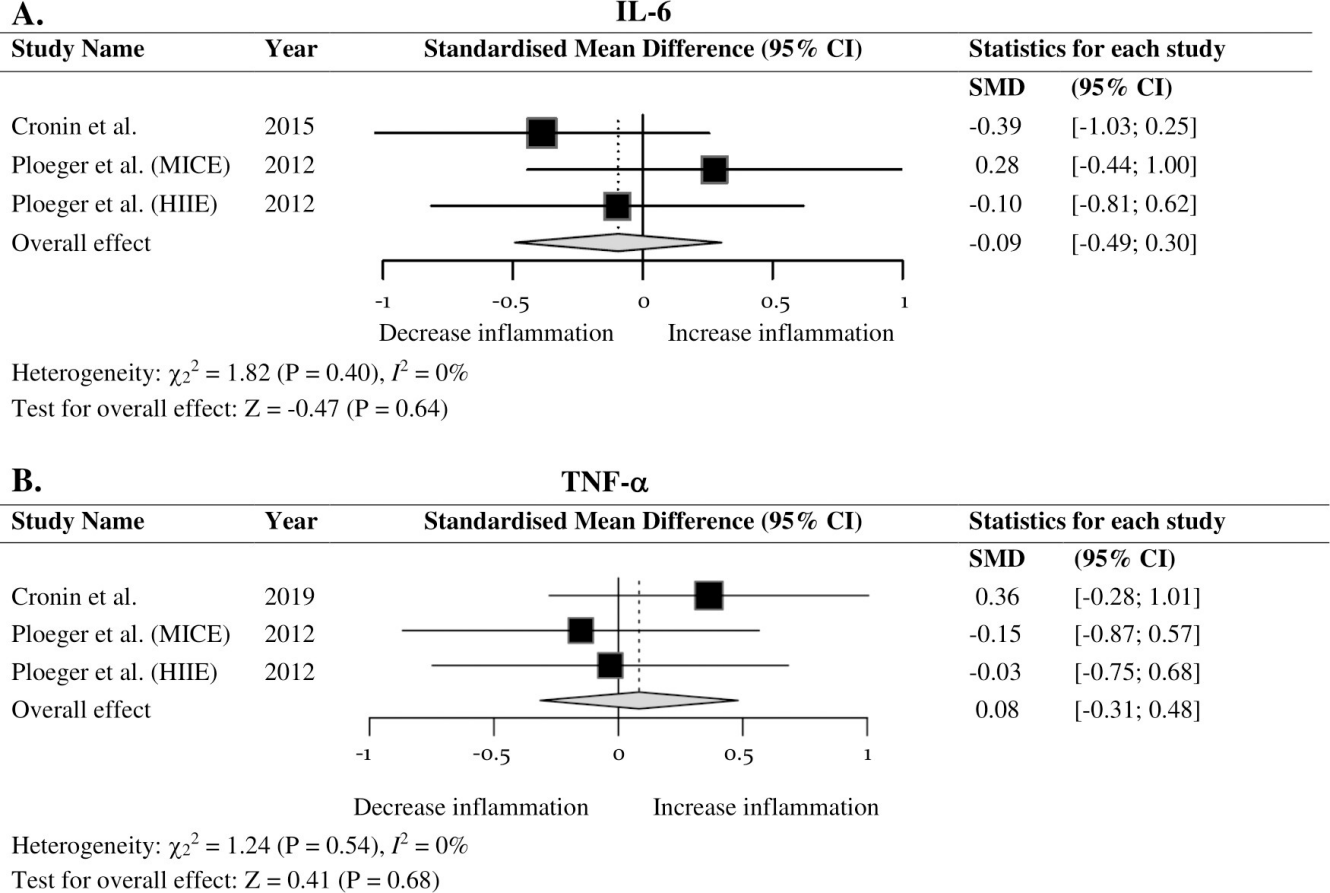
Forest plot of the combined effect of exercise on inflammatory markers in IBD. (A) IL–6. (B) TNF–α. Size of squares is proportional to weight of the study. Hedges’ *g* expressed as standardised mean difference (*SMD*) and 95% confidence interval (*CI*).

**Fig 3:**
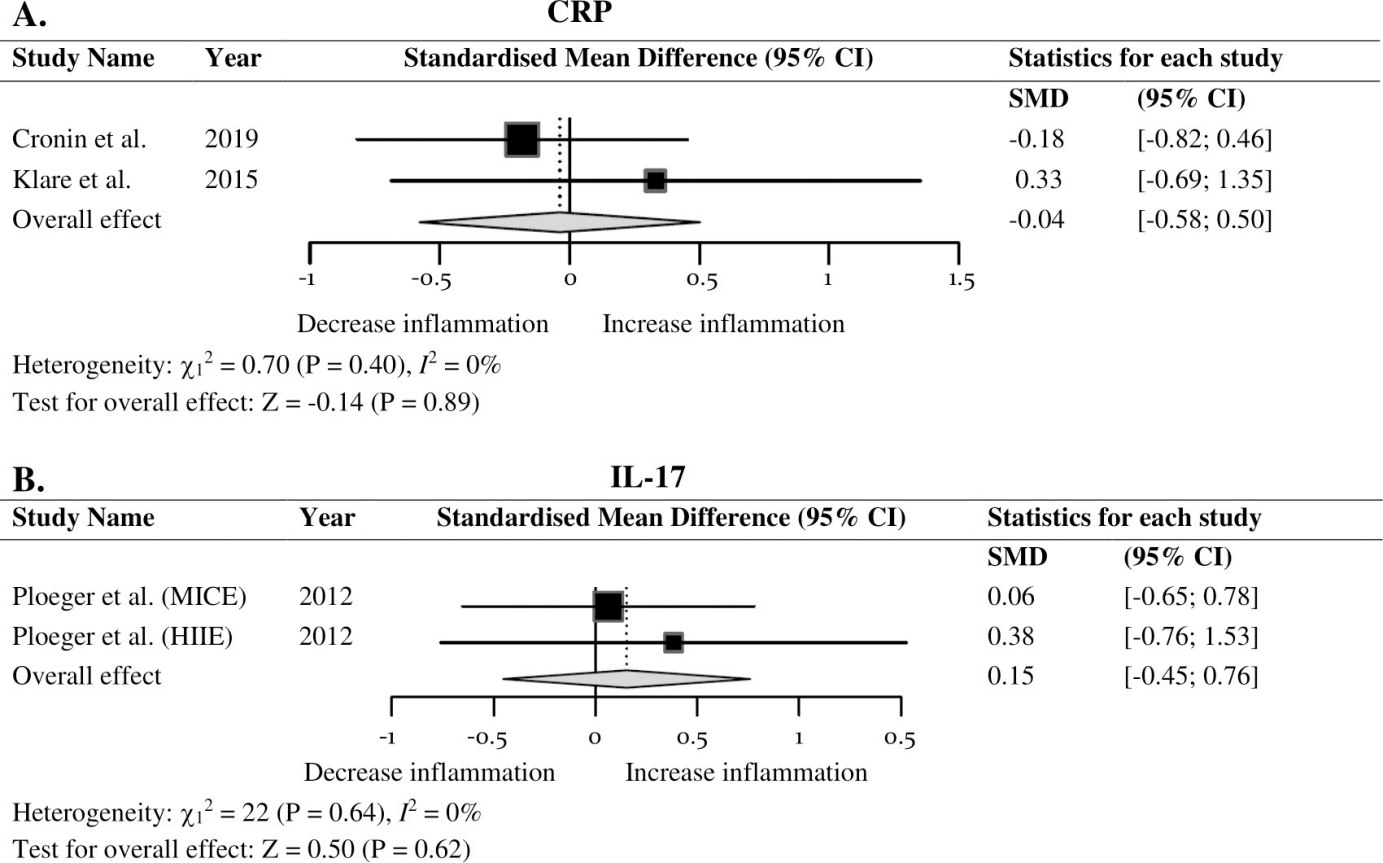
Forest plot of the combined effect of exercise on inflammatory markers in IBD. (A) CRP. (B) IL–17. Size of squares is proportional to weight of the study. Hedges’ *g* expressed as standardised mean difference (*SMD*) and 95% confidence interval (*CI*).

The inflammatory markers investigated were IL-6, TNF-α, CRP, IL-17, leukocytes, and lymphocytes. Two studies reported on IL-6 and TNF-α [45,46], with Ploeger et al. [46] completing two independent data sets which were extracted separately. Fig 2A demonstrates a forest plot examining the overall effect of exercise on IL-6. When all outcomes were collated for each trial, data found no significant differences (*SMD* = -0.09; 95% *CI* = -0.49, 0.30; *P* = 0.64). A similar trend for TNF-α was observed (*SMD* = 0.08; 95% *CI* = -0.31, 0.48; *P* = 0.68; Fig 2B). No heterogeneity was displayed in IL-6, TNF-α, C-reactive protein (CRP), or IL-17 (*I*^2^ = 0%). Further, there was no effect of exercise on CRP (*SMD* = -0.04; 95% *CI* = -0.58, 0.50; *P* = 0.89; Fig 3A). Ploeger et al. [46] was the only study to report on IL-17 and no significant overall effect of acute exercise was observed (*SMD* = 0.15; 95% *CI* = -0.45, 0.76; *P* = 0.62; Fig 3B). Fig A in S1 File displays a forest plot that presents the effect exercise has on leukocytes with no significant difference reported (*SMD* = 0.40; 95% *CI* = -0.53, 1.33; *P* = 0.40) and a high heterogeneity (*I*^2^ = 84%). Similarly, there was no effect of exercise on lymphocytes (*SMD* = 0.32; 95% *CI* = -0.33, 0.97; *P* = 0.33; Fig B in S1 File), with an identified small to moderate heterogeneity (*I*^2^ = 58%). Analysis was conducted on the inflammatory markers where two or more data sets were reported on, therefore, data extracted from Sharma et al. [61] was not utilised to complete the meta-analyses. Furthermore, data reported on neutrophils, monocytes, granulocytes, and growth factors, were not of interest for this meta-analysis; accordingly, no analyses were conducted.

## Discussion

The purpose of this systematic review and meta-analysis was three-fold. First, to investigate the anti-inflammatory response induced by acute and chronic exercise in IBD. Second, to ensure exercise is a feasible management strategy where the impacts of exercise on disease activity through associated symptoms were explored. Third, we examined the effect of different exercise modalities on the inflammatory response in IBD. To our knowledge, this is the first meta-analysis to examine the effectiveness of exercise interventions on the inflammatory response in IBD. These analyses revealed no overall significant effect of exercise on IL-6, TNF-α, CRP, IL-17, leukocytes, and lymphocytes with no adverse outcomes on symptomatic responses and favourable findings for the utilisation of mind-body techniques.

### Effect of exercise modalities on the inflammatory response

IL-6 possesses both pro- and anti-inflammatory properties and is associated with the pathological significance in IBD development, thus, is observed as a distinguishable mediator of intestinal inflammation [1,5,22]. Results from this meta-analysis revealed no overall pooled effect of exercise on IL-6 in IBD. Interestingly, Ploeger et al. [46] compared two acute interventions. The MICE intervention used an exercise intensity of 50% of their peak aerobic power and consisted of 2 sets of 30-minutes with a 6-minute break. Ploeger (MICE) reported a significant increase of IL-6 immediately post exercise in both IBD patients and healthy matched controls (*P* < 0.05). Similarly, Mendham et al. [63] reported a significant increase in IL-6 immediately post a 40-minute pre-determined moderate-to-vigorous intensity aerobic exercise bout (*P* < 0.01). The pre-determined 50% maximal aerobic workload utilised in Mendham et al. [63] is similar to the intensity utilised in Ploeger (MICE) intervention, thus, indicates the intensity may be sufficient to induce muscle-derived IL-6 response. However, the control group in the Ploeger et al. [46] study continued to experience a significant elevation in IL-6 from baseline to 60-minutes post exercise intervention (*P* < 0.05), as opposed to IBD participants who returned to pre-exercise levels in this time. The immunopathogenic nature of IBD and subsequent imbalances of the T-helper 1/T-helper 2 (Th1/Th2) paradigm further influences the stimulation of specific potent pro-inflammatory cytokines such as TNF-α [15]. This underlying mechanism may influence the acute exercise-induced inflammatory response. Specifically, the dysregulated response may overrule the prolonged effect of anti-inflammatory IL-6. To observe the extended IL-6 effect similar to healthy individuals, Ploeger et al. [46] exercise intervention may need to be longer in duration and/or of higher intensity.

Despite previous HIIE literature reporting significant increases in IL-6, no significant effects were found in the Ploeger et al. [46] intervention for IBD patients or for healthy matched controls. Such findings may be attributed to the HIIE protocol being insufficient in duration to induce inflammatory changes, 100% of peak aerobic power 15-seconds x 6 sets x 4 rounds. In comparison, Cullen et al. [64] found significant increases (*P* < 0.01) with three conditions of the same duration (35-minutes) and various intensities alternating between 50% and 80% of VO_2_max. The magnitude of the IL-6 response was significantly greater in the high-intensity intervention, 5 x 4-minute intervals at 80% VO_2_max interspersed with 3-minute intervals at 50% VO_2_max, compared to continuous 35-minutes at 50% of VO_2_max [64], suggesting the intervention implemented by Ploeger et al. [46], with a total of 6-minutes exercising, may not have been of sufficient duration to induce significant change in the abnormal IBD profile [15].

Further, while anti-inflammatory IL-6 increases acutely with exercise, chronic adaptations of IL-6 have shown inconsistent results. Goldhammer et al. [65] reported a significant reduction in IL-6 for people with coronary heart disease after a 12 week, 3 times per week, aerobic exercise training period. This intervention consisted of a continuous 45-minute bout of exercise on an ergometer (treadmill, rower, or stationary bike), at 70–80% of maximum heart rate, in addition to two 30-minute calisthenics exercise sessions weekly. Furthermore, Bruun et al. [66] reported a significant reduction of IL-6 following a 15-week moderate-intensity lifestyle intervention for morbidly obese individuals. Despite such findings, Cronin et al. [45] reported no change in IL-6 following an 8-week concurrent training program. The intervention involved moderate-intensity aerobic exercise with the progression based on the *couch to 5km* training program and resistance training that required a minimum 3 sets of 8 repetitions at 70% of one repetition maximum (1RM) [45]. Although the intensity was reported, the details of the duration of individual sessions were not. Similar non-significant results were observed following a 16-week concurrent training program for sedentary middle-aged men [67], suggesting further investigation is required regarding concurrent training for healthy individuals in advance of the connection to the inflammatory response in IBD.

High concentrations of TNF-α, as commonly seen in the pathogenesis of IBD and active disease states, possesses highly pleiotropic cellular effects that stimulate a cascade of endogenous mediators which direct host immunologic functions [68–70]. Following acute exercise, TNF-α is not commonly reported to increase in apparently healthy individuals [37]. Similarly, the current findings suggest no overall effect of TNF-α occurred in IBD patients, specifically in the Ploeger et al. [46] acute exercise interventions. The results reported in the Cronin et al. [45] training intervention are consistent with previous studies that completed 3 sessions per week for similar duration. For example, Allen et al. [71] reported no change in TNF-α after a 9-week intervention of either high-intensity interval training or prolonged intermittent sprint training. Furthermore, Mendham et al. [72] reported no difference in TNF-α following either moderate-intensity cycling or small-sided games (SSG) after 8-weeks. The influence of different exercise modalities on TNF-α requires further investigation to sufficiently understand the mechanisms responsible, preceding in connection to the inflammatory abnormalities in IBD.

Acute phase protein, CRP, is a well-established marker in IBD [23,24]. The reduction of CRP in longitudinal studies may indicate regular training is advantageous to reduce low-grade inflammation [38]. Specific studies identified significant decreases of CRP over a 12-month moderate-intensity regular training period [73]. In contrast to previous findings, no pooled outcome of CRP in IBD patients occurred over a regular exercise period. The 8 to 10-week duration of the exercise interventions utilised by Cronin et al. [45] and Klare et al. [31], respectively, could have potentially decreased CRP as Mendham et al. [72] reported a significant decrease in CRP in both SSG’s and a cycling protocol in only 8-weeks. However, in contrast to Mendham et al. [72], the studies in the meta-analyses did not objectively assess moderate-intensity, thus, increasing the within study variance. Furthermore, similar to Cronin et al. [45], Klare et al. [31] did not report on duration of individual weekly sessions. Such limitations in study design may impact overall findings in this review.

The pro-inflammatory cytokine IL-17 is speculated to play a major role in the destructive inflammation observed in IBD, specifically through the upregulation of additional pro-inflammatory cytokines, such as TNF-α, IL-1β, and IL-6 [1,22]. However, the source and cause of the IL-17 response after exercise is less understood in healthy individuals [74]. In the study conducted by Ploeger et al. [46] the relationship of IL-17 and acute exercise in IBD was explored, although can be assumed to be under-powered as IL-17 was only detectable in less than half of the IBD participants. Findings from this meta-analysis suggest no effect on IL-17, following acute MICE and HIIE. Similarly, Dorneles et al. [75] study utilised moderate-intensity interval exercise and an additional HIIE intervention found no significant differences in systemic concentrations of IL-17 in either group. Conversely, Sugama et al. [76] recorded a significant decrease in IL-17 after exhaustive endurance exercise in male triathletes. This suggests that the intensity and duration of the Ploeger et al. [46] MICE intervention may not have been sufficient to elicit a change in IL-17. There is limited research available on the effects of regular training on IL-17 in the IBD population, therefore this parameter warrants further investigation.

### Effect of exercise on disease activity and symptoms

The findings of the meta-analyses suggest that regardless of differing intensity and duration, exercise produces immune and inflammatory responses in IBD. The exercise interventions examined may be insufficient to induce significant overall changes in inflammatory markers in IBD participants. Accordingly, avert the subsequent favourable reduction in disease activity and symptoms. However, Elsenbruch et al. [60] reported a significantly greater improvement in the IBDQ bowel symptoms component following a mind-body intervention that involved a series of stress management and moderate-intensity exercise techniques. Similarly, Sharma et al. [61] concluded fewer patients reported arthralgia, whilst more control participants reported colicky pain, following an 8-week yoga-based intervention; however, no difference was reported to be significant. These findings were also supported by Gerbarg et al. [77] who conducted a breath-body-mind workshop for IBD patients that reported significant within-group comparisons between baseline, week 6, and week 26 for improvements in IBD symptoms (*P* = 0.02; *P* = 0.04) and IBDQ (*P* = 0.01; *P* = 0.01), as well as perceived stress at 26 weeks (*P* = 0.01). The improvements of IBD symptoms may be attributed to the reduction of psychological stress and subsequent modification to the HPA axis. A wide range of psychological aspects are known to alter the function of the gastrointestinal tract [78], through the disruption of the gut-brain axis, in particular the HPA axis [11,79]. Typically, glucocorticoids, a known primary mediator of the HPA axis, has an essential role in negative feedback regulation of the inflammatory response by either inhibiting pro-inflammatory cytokines or promoting the production of anti-inflammatory cytokines [80–82]. However, a disruption of the inflammatory response in the HPA axis can transpire by psychological stress. The stress-induced activation of the HPA axis enhances the secretion of neuroendocrine mediators; this activates lower-affinity [83] and consequently diminishes immune cell sensitivity [28,80]. These known immunological and neuroendocrine changes result in an imbalance of pro- and anti-inflammatory cytokines, contribute to intestinal inflammation, and enhance IBD exacerbation [28,80,84–86]. Therefore, the possible improvement of the HPA axis has the potential to ameliorate abnormal inflammatory responses experienced in IBD. Stabilisation of a dysregulated inflammatory and stress response may be achieved by regular exercise and attributed towards the reduction in inflammatory cytokines and stress hormones [87]. Furthermore, IBD symptoms may be reduced by interventions that alleviate psychological distress such as stress management [60], yoga [61], and other mind-body techniques [77]. Although this review did not show significant improvements in the inflammatory mediators, these findings suggest reductions in psychological stress is an important aspect in IBD to reduce intestinal inflammation and disease exacerbation.

### Limitations

To date, published research in this area is sparse. Five studies met the eligibility criteria; therefore, one limitation of this review is that the small sample size result in limited sensitivity of the analysis and no assessment of publication bias. Furthermore, the quality assessment of individual studies was affected by the inability to blind participants, subjects, or therapists, however, it was further affected by poor blinding of the outcome assessors in a number of interventions. Only citations accessible in English were considered in this review and it is therefore possible that additional data in other languages may have been overlooked. Finally, a major limitation is the marked heterogeneity among the identified literature, which involved measurement of a range of different inflammatory markers and completion of various exercise interventions. Future replication of the meta-analysis should consider the different inflammatory entities of IBD (CD and UC), distinguish between acute and chronic exercise interventions, and separate paediatric and adult patients.

## Conclusion

In conclusion, this study investigated the current literature that has evaluated the effects of exercise on the inflammatory response for people with IBD. Overall, there were no significant differences in the inflammatory markers analysed following exercise intervention in people with IBD. Whilst this may indicate that exercise plays no role in improving the inflammatory profile of IBD sufferers, it may also reflect the paucity of available research and the marked heterogeneity of exercise interventions in the research that is available. The studies eligible for this meta-analysis limits the reliability of the results, however it emphasises the need for additional research in this domain. A greater insight into the exercise-induced inflammatory response in IBD is required to make definitive conclusions; more explicit IBD markers and specific inflammatory markers suitable for exercise are necessary to establish distinct outcomes. Furthermore, research into specific exercise modalities is necessary to produce exercise guidelines for implementation into clinical settings for IBD patients.

Despite no significant inflammatory changes, no adverse effects were reported on IBD associated symptoms following exercise which suggests exercise participation is safe for IBD patients and is unlikely to exacerbate their disease. The review revealed favourable symptomatic outcomes associated with regular exercise such as yoga, as well as a combination of moderate exercise and mind-body techniques such as stress management. Further research with adequate inflammatory markers is required to make definitive conclusions. Research with different exercise modalities and the incorporation of mind-body techniques should be investigated to elucidate the effects from acute and chronic exercise interventions on acute inflammatory responses and systemic inflammation. This information may establish the effectiveness of exercise for the IBD population as well as possibly determine exercise recommendations, create clinical guidelines, and provide an alternative and/or complementary treatment strategy for IBD sufferers.

## Supporting information

S1 Table PRISMA 2020 checklist. (PDF)

S1 File Forest plot of the combined effect of exercise on inflammatory markers in IBD. (A) Leukocytes. (B) Lymphocytes. Size of squares is proportional to weight of the study. Hedges’ *g* expressed as standardised mean difference (*SMD*) and 95% confidence interval (*CI*).
(PDF)

S1 Dataset Dataset of meta-analyses. The calculated within-group effect size expressed as Hedges’ *g* and standard error.
(XLSX)
