# Supplementary material for: The exercise-induced inflammatory response in inflammatory bowel disease: A systematic review and meta-analysis
Source: PLoS One. 2022 Feb 4;17(2):e0262534. doi: 10.1371/journal.pone.0262534 (PMC8815877; doi:10.1371/journal.pone.0262534)
Supplement: S1 File — (A) Leukocytes. (B) Lymphocytes. Size of squares is proportional to weight of the study. Hedges’ g expressed as standardised mean difference (SMD) and 95% confidence interval (CI). (PDF) [file pone.0262534.s002.pdf]

A.

Leukocytes

Study Name	Year	SMD	(95% CI)
Elsenbruch et al.	2005	0.14	[-0.58; 0.85]
Klare et al.	2015	-0.72	[-1.46; 0.02]
Ploeger et al. (MICE)	2012	1.66	[0.83; 2.50]
Ploeger et al. (HIIE)	2012	0.58	[-0.15; 1.31]
Overall effect		0.40	[-0.53; 1.33]

Standardised Mean Difference (95% CI) — **Statistics for each study**

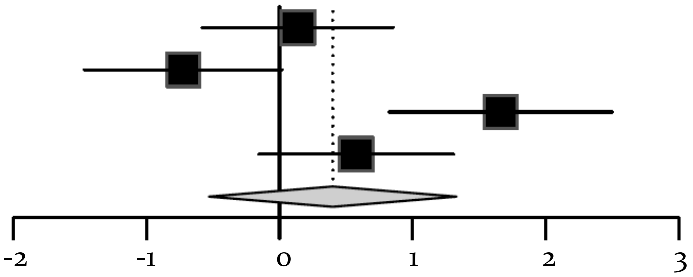

Decrease inflammation Increase inflammation

Heterogeneity: $\chi_3^2 = 18.25$ ($P < 0.001$), $I^2 = 84\%$ [-0.53; 1.33]

Test for overall effect: Z = 0.84 (P = 0.40)

B.

Lymphocytes

Study Name	Year	SMD	(95% CI)
Elsenbruch et al.	2005	-0.11	[-0.82; 0.0.61]
Ploeger et al. (MICE)	2012	1.00	[0.24; 1.76]
Ploeger et al. (HIIE)	2012	0.11	[-0.61; 0.82]
Overall effect		0.32	[-0.33; 0.97]

Standardised Mean Difference (95% CI) — **Statistics for each study**

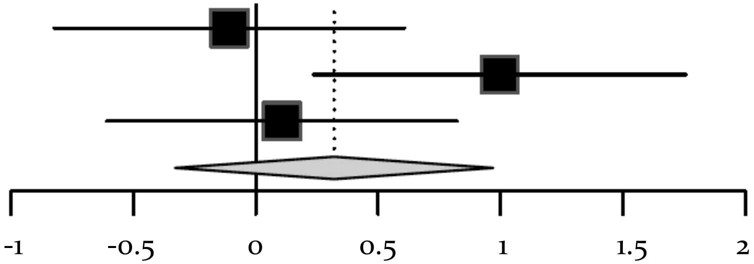

Decrease inflammation Increase inflammation

Heterogeneity: $\chi_2^2 = 4.75$ ($P = 0.09$), $I^2 = 58\%$

Test for overall effect: Z = 0.97 (P = 0.33)
